# Low dialysate sodium and 48-h ambulatory blood pressure in patients with intradialytic hypertension: a randomized crossover study

**DOI:** 10.1093/ndt/gfae104

**Published:** 2024-05-06

**Authors:** Fotini Iatridi, Konstantinos Malandris, Robert Ekart, Efstathios Xagas, Antonios Karpetas, Marieta P Theodorakopoulou, Artemios Karagiannidis, Areti Georgiou, Aikaterini Papagianni, Pantelis Sarafidis

**Affiliations:** First Department of Nephrology, Hippokration Hospital, Aristotle University of Thessaloniki, Thessaloniki, Greece; Clinical Research and Evidence-Based Medicine Unit, Aristotle University of Thessaloniki, Thessaloniki, Greece; Department of Nephrology, University Clinical Centre Maribor, Maribor, Slovenia; Frontis Dialysis Center, Athens, Greece; Therapeutiki Hemodialysis Unit, Thessaloniki, Greece; First Department of Nephrology, Hippokration Hospital, Aristotle University of Thessaloniki, Thessaloniki, Greece; First Department of Nephrology, Hippokration Hospital, Aristotle University of Thessaloniki, Thessaloniki, Greece; First Department of Nephrology, Hippokration Hospital, Aristotle University of Thessaloniki, Thessaloniki, Greece; First Department of Nephrology, Hippokration Hospital, Aristotle University of Thessaloniki, Thessaloniki, Greece; First Department of Nephrology, Hippokration Hospital, Aristotle University of Thessaloniki, Thessaloniki, Greece

**Keywords:** ABPM, blood pressure, dialysate sodium, hemodialysis, intradialytic hypertension (IDH)

## Abstract

**Background:**

Intradialytic hypertension (IDH) is associated with increased risk for cardiovascular events and mortality. Patients with IDH exhibit higher 48-h blood pressure (BP) levels than patients without this condition. Volume and sodium excess are considered a major factor contributing in the development of this phenomenon. This study evaluated the effect of low (137 mEq/L) compared with standard (140 mEq/L) dialysate sodium concentration on 48-h BP in patients with IDH.

**Methods:**

In this randomized, single-blind, crossover study, 29 patients with IDH underwent four hemodialysis sessions with low (137 mEq/L) followed by four sessions with standard (140 mEq/L) dialysate sodium, or vice versa. Mean 48-h BP, pre-/post-dialysis and intradialytic BP, pre-dialysis weight, interdialytic weight gain (IDWG) and lung ultrasound B-lines were assessed.

**Results:**

Mean 48-h systolic/diastolic BP (SBP/DBP) were significantly lower with low compared with standard dialysate sodium concentration (137.6 ± 17.0/81.4 ± 13.7 mmHg with low vs 142.9 ± 14.5/84.0 ± 13.9 mmHg with standard dialysate sodium, *P* = .005/*P* = .007, respectively); SBP/DBP levels were also significantly lower during the 44-h and different 24-h periods. Low dialysate sodium significantly reduced post-dialysis (SBP/DBP: 150.3 ± 22.3/91.2 ± 15.1 mmHg with low vs 166.6 ± 17.3/94.5 ± 14.9 mmHg with standard dialysate sodium, *P* < .001/*P* = .134, respectively) and intradialytic (141.4 ± 18.0/85.0 ± 13.4 mmHg with low vs 147.5 ± 13.6/88.1 ± 12.5 mmHg with standard dialysate sodium, *P* = .034/*P* = .013, respectively) BP compared with standard dialysate sodium. Pre-dialysis weight, IDWG and pre-dialysis B lines were also significantly decreased with low dialysate sodium.

**Conclusions:**

Low dialysate sodium concentration significantly reduced 48-h ambulatory BP compared with standard dialysate sodium in patients with IDH. These findings support low dialysate sodium as a major non-pharmacologic approach for BP management in patients with IDH.

**Trial registration:**

ClinicalTrials.gov study number NCT05430438.

## Graphical Abstract

**Graphical Abstract:**
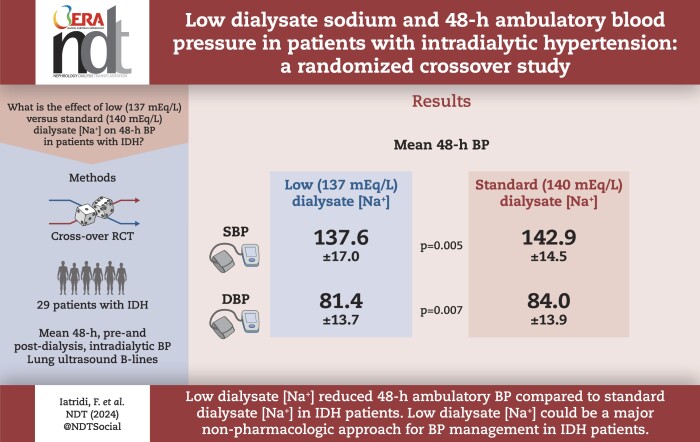

KEY LEARNING POINTS **What was known:**

- Patients with intradialytic hypertension (IDH) exhibit higher blood pressure (BP) levels during the interdialytic interval compared with patients without; IDH is associated with increased risk for cardiovascular disease and mortality.
- Previous studies showed lower intradialytic and post-dialysis BP with lower dialysate sodium concentration, but none assessed the effect on 48-h ambulatory BP.

**This study adds:**

- This is the first study examining the effect of low dialysate sodium concentration on 48-h ambulatory BP in patients with IDH.
- Mean 48-h systolic/diastolic BP were significantly lower with low compared with standard dialysate sodium concentration.

**Potential impact:**

- Low dialysate sodium concentration resulted in significantly lower 48-h ambulatory BP levels compared with standard dialysate sodium in patients with IDH.
- These findings support the use of low dialysate sodium as a non-pharmacologic strategy for BP management in patients with IDH.

## INTRODUCTION

Hypertension is the most common risk factor for cardiovascular disease in patients with end-stage kidney disease (ESKD) undergoing hemodialysis with an estimated prevalence around 85%, and is associated with increased risk for cardiovascular events [1].

Sodium and water removal during the hemodialysis session represents a major determinant of blood pressure (BP) fluctuations in ESKD; as such, the usual trajectory of BP levels through the intra- and interdialytic interval is a decrease in BP during hemodialysis, as a response to ultrafiltration, followed by a more gradual increase during the interdialytic interval [1–3]. However, apart from this specific pattern of BP change during dialysis, a smaller subset of patients experience a diverse response, i.e. a “paradoxical” rise in BP during or immediately after dialysis which is usually referred to as intradialytic hypertension (IDH) [4, 5].

Over the course of the last decade, accumulating evidence demonstrated that IDH is a phenomenon that occurs in approximately 10%–15% of the dialysis population, while patients with IDH exhibit an increased risk for cardiovascular events, cardiovascular and all-cause mortality, compared to patients exhibiting the usual pattern of BP fall during dialysis [6–10]. The pathophysiological background of this condition is multifactorial and several mechanisms have been proposed to be implicated in the development of IDH. Among them, volume and sodium overload are listed among the important contributors to the pathogenesis of the phenomenon [4, 11, 12].

Previous studies in patients with IDH attempted to decrease intradialytic sodium gain and subsequently BP rise through modification of dialysate sodium. In a randomized crossover study in 16 patients with IDH, low dialysate sodium concentration was associated with lower intradialytic systolic BP (SBP) over a 1-week period compared with high (5 mEq/L higher than serum sodium) dialysate sodium [13]. A non-randomized study examined the effect of low (136 mEq/L) compared with standard (140 mEq/L) dialysate sodium on peridialytic and intradialytic BP, with similar results [14]. The only previous study using 24-h ambulatory blood pressure monitoring (ABPM), evaluated 11 patients with IDH to assess the effect of individualized isonatremic vs hyponatremic vs standard dialysate sodium of 140 mEq/L [15], showing a reduction in 24-h BP during the hyponatremic phase.

Several lines of evidence suggest that in hemodialysis patients 48-h BP recordings over the intra- and interdialytic interval display stronger associations with adverse outcomes [16, 17]. Previous observations from our group suggest persistently elevated BP levels during the whole 48-h interval in patients with IDH compared to hemodialysis patients without the phenomenon [18]. In a recent study, we also observed that the association of IDH with adverse clinical outcomes is modified by 48-h BP levels, suggesting that the high cardiovascular risk in this population is at least partially attributed to the persistently elevated interdialytic BP [19]. As such, the aim of this study was to examine the effect of low (137 mEq/L) compared to standard (140 mEq/L) dialysate sodium on ambulatory BP during a complete 48-h intra and interdialytic period in patients with IDH, using a randomized crossover design.

## MATERIALS AND METHODS

### Study participants

This is a multicenter, prospective, randomized, single-blind, crossover study aiming to examine the effect of low versus standard dialysate sodium concentration on 48-h ambulatory BP. Patients were recruited from four Hemodialysis Units, three located in Greece and one in Slovenia, between June 2022 and June 2023. All centers participating in the study followed local practice that included use of dialysate sodium concentration of 140 mEq/L. Inclusion criteria were: (i) adult patients with ESKD treated with a standard thrice-weekly hemodialysis schedule for >3 months; (ii) IDH, defined as SBP rise ≥10 mmHg from pre- to post-dialysis in at least four out of six consecutive hemodialysis sessions; (iii) patients at dry weight, as assessed by clinical criteria; and (iv) informed written consent. Exclusion criteria were: (i) patients with post-dialysis SBP <130 mmHg in at least four out of six consecutive hemodialysis treatments during the selection period prior to study enrollment; (ii) presence of old, non-functional, arteriovenous fistula in the arm used for ABPM that could interfere with proper ABPM recording; (iii) contraindications for receiving the intervention of low dialysate sodium (e.g. frequent hypotensive episodes requiring fluid resuscitation); (iv) pre-dialysis serum sodium <130 mEq/L or >142 mEq/L at recruitment; (v) modification of dry weight or antihypertensive treatment during 1 month prior to enrollment; (vi) history of seizures or dialysis disequilibrium syndrome; (vii) hospitalization for any cause during 1 month prior to enrollment; and (viii) active malignancy or other comorbidities with poor prognosis.

The study protocol was reviewed and approved by the Ethics Committee of the School of Medicine, Aristotle University of Thessaloniki and all procedures were performed according to the Declaration of Helsinki (2013 Amendment). The trial is registered in ClinicalTrials.gov (NCT05430438).

### Study procedures

Patients were assessed for eligibility based on records of peridialytic BP measurements of a 2-week period to confirm the presence of IDH. Demographic and anthropometric characteristics, medical history, medication, comorbidities and other dialysis-related parameters were recorded. Following enrollment, all participants had a baseline evaluation performed before a mid-week dialysis session, when physical examination and venous blood sampling for routine laboratory testing were performed. Hydration status was assessed with lung ultrasound (US); peridialytic and intradialytic BP was assessed with the Mobil-O-Graph device; pre- and post-dialysis weight was also recorded. After baseline evaluation, patients were randomly assigned to two groups, which received the two interventions in the opposite order. The first group (Group A) received low, i.e. 137 mEq/L, dialysate sodium followed by standard, i.e. 140 mEq/L dialysate sodium concentration and the second (Group B) standard dialysate sodium followed by low dialysate sodium concentration.

All participants underwent 1 week after baseline evaluation on standard dialysate sodium. Thereafter, participants underwent four hemodialysis sessions, starting from a mid-week session (i.e. Wednesday or Thursday), with low or standard dialysate sodium concentration, depending on the randomization arm. Before the start of the fourth session, patients were assessed with lung US and the 48-h ABPM started. Interdialytic weight gain (IDWG), pre- and post-dialysis weight and other dialysis-related parameters were also documented. A 2-week washout period with standard dialysate sodium of 140 mEq/L followed for both groups. Finally, participants underwent another set of four hemodialysis sessions with the opposite dialysate sodium concentration. Before the start of the fourth session the above procedures were repeated.

During the study ultrafiltration volume was determined based on the participants’ prespecified dry weight, defined according to standard clinical criteria. Dry weight, antihypertensive medication, epoetin administration and parameters related to dialysate composition—apart from dialysate sodium—were not allowed to change during the course of the study. Participants were instructed to continue their usual daily activities, including physical activity, sleep, food and water intake, and follow their medication with no deviations over the study period.

### Assessments

ABPM was performed with the Mobil-O-Graph device (IEM, Stolberg, Germany), an automated oscillometric device previously validated for brachial BP measurement according to standard protocols [20] and shown to provide identical values with a widely used ABPM monitor [21]. The recording started before the fourth session of each period, which was a mid-week dialysis session and was scheduled to last for a complete standard 48-h intra- and interdialytic period. The device was fitted on the non-fistula arm with appropriate-size cuffs and was programmed to measure BP every 20 min during day-time (07:00 to 22:59) and every 30 min during night-time (23:00 to 06:59). BP measurements during the hemodialysis session were recorded with the ABPM device at the aforementioned intervals. Recordings were included in the analysis if >80% of the total readings were valid and a maximum of two non-consecutive day-hours had fewer than two valid measurements and a maximum of one night-hour had no valid reading. Patients with invalid recordings remained on the same dialysate sodium concentration and repeated the ABPM 1 week later. In order to minimize the possible effect of manually obtained BP measurements, only measurements recorded at the pre-specified time intervals at which the device was set to measure BP were used in this analysis. Peri- and intra-dialytic BP were also recorded with the Mobil-O-Graph device. For peri-dialytic readings, patient remained in the sitting position in their dialysis chair for at least 5 min before and after hemodialysis session.

Lung US was performed with the portable VScan device (GE Healthcare, Horten, Norway) which has shown to provide reliable high-resolution images of the lungs and heart, similar to those obtained with bigger steady US devices [22]. We performed the most widely used technique which involves scanning of a total of 28 sites in both lungs, with the patient in a lying position [23, 24]. The transducer was placed vertically along the parasternal, mid-clavear, anterior axillary and mid-axillary lines, from the second to the fifth intercostal space on the right and from the second to the fourth on the left side. The total number of US-B lines on each site was recorded.

### Study endpoints

The primary endpoint was the difference in 48-h SBP between low and standard dialysate sodium concentration. Secondary endpoints included 48-h DBP, pre- and post-dialysis and intradialytic SBP/DBP, SBP/DBP over different time periods of the 48-h recording (Supplementary data, Fig. S1), pre-dialysis weight, IDWG and US-B lines. The differences in the change (Delta, Δ) in pre-dialysis, post-dialysis and intradialytic BP from baseline between low and standard dialysate sodium were also included in secondary outcomes.

### Sample size, randomization and blinding

Sample size calculation was performed with Hedwig software of Massachusetts General Hospital Biostatistics Center [25]. It was determined that 28 participants were needed to detect, with 80% power and level of significance α = 0.05, a 6 mmHg difference in 48-h SBP between low versus standard dialysate sodium concentrations, assuming a standard deviation (SD) of 10 mmHg between the two periods, in a pairwise crossover design. A total of 30 patients were recruited to count in for a potential dropout of 8%. Block randomization was used to determine treatment order based on a computer-generated randomization list. Participants were blinded to intervention order, but providers and assessors were unblinded.

### Statistical analysis

Statistical analysis was performed with Statistical Package for Social Sciences version 22.0 (SPSS Inc., Chicago, IL, USA). Continuous variables are presented as mean ± SD or median and interquartile range (Q1–Q3) depending on the normality of distribution, assessed with Shapiro–Wilk test. Categorical variables are expressed as frequencies and percentages (*n*, %). Paired Student’s *t*-test or Wilcoxon's signed rank test were used, according to the normality of distribution, to examine differences in BP and BP change from baseline, pre-dialysis weight, IDWG and US-B lines between low versus standard dialysate sodium. Bivariate correlation coefficients (r) were calculated using the Pearson's product formula. A *P*-value <.05 (two-tailed) was considered statistically significant in all comparisons.

## RESULTS

### Baseline characteristics

**Figure 1:**
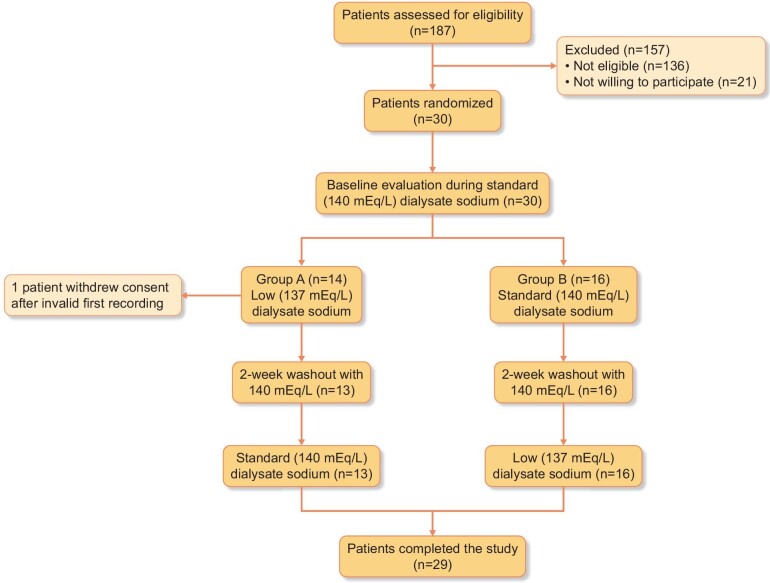
Study flowchart.

The flowchart of study participants is shown in Fig. 1. From a total of 187 assessed, 30 participants fulfilled the inclusion/exclusion criteria and were included in the study. Among patients fulfilling all the inclusion criteria, three patients were excluded due to modification of dry weight or antihypertensive medication during 1 month before enrolment. Fourteen patients were randomized to Group A and 16 to Group B. Among them, nine participants had to repeat one measurement due to an invalid recording; three of them repeated the ABPM during the standard dialysate sodium phase and five during the low dialysate sodium phase. One patient randomized to Group A with an invalid first 48-h ABPM refused to repeat the recording and withdrew consent. Thus, a total of 29 patients (17 male and 12 female) completed the study and are included in the analysis.

Baseline demographic, clinical, laboratory and dialysis-related parameters of the study population are presented in Table 1. Participants had a mean age of 64.6 ± 17.6, and median hemodialysis vintage 28.7 (8.3–69.8) months. With regards to major comorbidities, all participants were hypertensive, around 40% of them had diabetes mellitus and 40% coronary heart disease. The most common cause of ESKD was diabetic kidney disease followed by hypertensive kidney disease. As shown in Table 1, the most commonly prescribed drug classes were calcium channel blockers (CCBs) and β-blockers. Seventeen patients (58.6%) were receiving loop diuretics and had a reported residual diuresis of 0.4 ± 0.3 L/24 h. With regards to peri- and intradialytic BP, mean pre-dialysis BP was 148.8 ± 19.9/87.3 ± 13.5 mmHg, whereas mean post-dialysis BP was 167.3 ± 20.2/96.8 ± 17.8 mmHg; mean intradialytic BP at baseline evaluation was 148.7 ± 18.2/88.1 ± 13.0 mmHg.

**Table 1:** Baseline demographic, clinical and laboratory characteristics of the study population.

**Parameter**	**Value**
*N*	29
Age (years)	64.6 ± 17.6
Male gender (*n*, %)	17 (58.6)
Dry weight (kg)	68.97 ± 15.41
BMI (kg/m^2^)	24.58 ± 4.78
Dialysis vintage (months)	28.70 (8.3, 69.8)
Randomization group^a^ (*n*, %)	
Group A	13 (44.8)
Group B	16 (55.2)
Comorbidities (*n*, %)	
Hypertension	29 (100)
Diabetes mellitus	12 (41.4)
Dyslipidemia	29 (100)
Coronary heart disease	11 (37.9)
Heart failure	12 (41.4)
Peripheral vascular disease	9 (31.0)
Smoking	13 (44.8)
Primary cause of ESKD (*n*, %)	
Diabetic kidney disease	7 (24.1)
Hypertension or ischemic renal disease	5 (17.2)
Glomerulonephritis	3 (10.3)
Inherited diseases	1 (3.4)
Tubulointerstitial nephritis	2 (6.9)
Other	1 (3.4)
Unknown	10 (34.5)
Laboratory values	
Hemoglobin (g/dL)	10.90 ± 1.18
Serum urea (mg/dL)	119.5 [94.3–142.5]
Serum creatinine (mg/dL)	7.99 ± 2.35
Serum sodium (mEq/L)	137.5 ± 3.2
Serum potassium (mEq/L)	5.11 ± 0.82
Serum calcium (mg/dL)	8.91 ± 0.70
Serum phosphate (mg/dL)	4.37 ± 1.32
Serum albumin (g/dL)	3.97 ± 0.37
Parathormone (pg/mL)	271.20 (115.85–398.00)
Antihypertensive medication (*n*, %)	
Number of drugs	
0 drugs	3 (10.3)
1 drug	3 (10.3)
2 drugs	6 (20.7)
3 drugs	6 (20.7)
4 drugs	6 (20.7)
≥5 drugs	5 (17.2)
Type of drugs	
ACEIs	1 (3.4)
ARBs	10 (34.5)
CCBs	18 (62.1)
β-blockers	18 (62.1)
Loop diuretics	17 (58.6)
Centrally active agents	5 (17.2)
α blockers	11 (37.9)
ESA treatment (*n*, %)	25 (83.3)
IDWG (kg)	1.7 ± 0.91
Pre-dialysis weight (kg)	70.68 ± 15.83
UF rate (L/h)	0.48 ± 0.24
Pre-dialysis SBP (mmHg)	148.8 ± 19.9
Pre-dialysis DBP (mmHg)	87.3 ± 13.5
Post-dialysis SBP (mmHg)	167.3 ± 20.2
Post-dialysis DBP (mmHg)	96.8 ± 17.8
Intradialytic SBP (mmHg)	148.7 ± 18.2
Intradialytic DBP (mmHg)	88.1 ± 13.0
US-B lines	8 (5–13)

Data are presented as mean ± SD, median (interquartile range) or *n* (%).

^a^Group A received 137 mEq/L followed by 140 mEq/L dialysate sodium Group B received 140 mEq/L followed by 137 mEq/L dialysate sodium.

ACEIs, angiotensin-converting enzyme inhibitors; ARBs, angiotensin II receptor blockers; BMI, body mass index; CCBs, calcium channel blockers; ESA, erythropoietin-stimulating agent; UF, ultrafiltration.

### Levels of 48-h ambulatory BP, peridialytic and intradialytic BP

**Figure 2:**
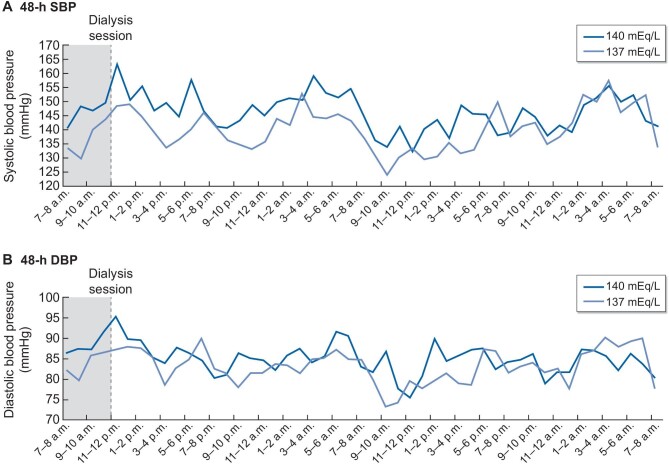
BP trajectories with low and standard dialysate sodium concentration during the 48-h period for (**A**) SBP and (**B**) DBP (data displayed for patients in morning shift).

The levels of 48-h, peri- and intradialytic BP with the two different dialysate sodium concentrations are depicted in Table 2. Mean 48-h SBP was significantly lower with low compared with standard dialysate sodium concentration (137.6 ± 17.0 mmHg with low vs 142.9 ± 14.5 mmHg with standard dialysate sodium, *P* = .005). Similarly, 48-h DBP was significantly lower with low dialysate sodium (81.4 ± 13.7 mmHg with low vs 84.0 ± 13.9 mmHg with standard dialysate sodium, *P* = .007). Figure 2 portrays the trajectories of SBP/DBP with low and standard dialysate sodium during the whole 48-h period, highlighting the significantly lower BP levels observed with the lower dialysate sodium concentration.

**Table 2:** Ambulatory BP levels during the 44-h interval and the respective 24-h periods with low vs standard dialysate sodium.

**Parameter**	**Low dialysate sodium**	**Standard dialysate sodium**	***P***
48-h SBP (mmHg)	137.6 ± 17.0	142.9 ± 14.5	**.005**
48-h DBP (mmHg)	81.4 ± 13.7	84.0 ± 13.9	**.007**
Pre-dialysis SBP (mmHg)	138.7 ± 14.3	146.4 ± 17.9	.087
Pre-dialysis DBP (mmHg)	83.7 ± 11.9	86.5 ± 10.6	.135
Post-dialysis SBP (mmHg)	150.3 ± 22.3	166.6 ± 17.3	**<.001**
Post-dialysis DBP (mmHg)	91.2 ± 15.1	94.5 ± 14.9	.134
Intradialytic SBP (mmHg)	141.4 ± 18.0	147.5 ± 13.6	**.034**
Intradialytic DBP (mmHg)	85.0 ± 13.4	88.1 ± 12.5	**.013**
44-h SBP (mmHg)	137.2 ± 17.4	142.4 ± 15.4	**.01**
44-h DBP (mmHg)	81.0 ± 14.0	83.5 ± 14.5	**.014**
First 24-h SBP (mmHg)	137.5 ± 18.2	143.9 ± 15.4	**.001**
First 24-h DBP (mmHg)	81.8 ± 14.0	84.4 ± 14.4	**.005**
First 20-h SBP (mmHg)	136.6 ± 19.6	142.9 ± 17.5	**.004**
First 20-h DBP (mmHg)	80.9 ± 14.6	83.4 ± 15.6	**.018**
Second 24-h SBP (mmHg)	137.9 ± 16.0	141.8 ± 14.7	.076
Second 24-h DBP (mmHg)	81.0 ± 13.8	83.6 ± 13.9	**.034**
IDWG (kg)	1.4 ± 0.8	1.8 ± 0.7	**.012**
Pre-dialysis body weight (kg)	70.7 ± 16.1	71.1 ± 16.2	**.024**
US-B lines	7 (4–14)	8 (6–13)	**.036**

Data are presented as mean ± SD or median (interquartile range).

The 44-h BP parameters represent the 48-h period minus the 4-h dialysis session.

First 20-h BP parameters represent the first 24-h period minus the 4-h dialysis session.

UF, ultrafiltration.

Pre-dialysis SBP/DBP were numerically lower with low dialysate sodium, but these differences were not statistically significant. Importantly, post-dialysis and intradialytic SBP both showed significant differences between low and standard dialysate sodium (post-dialysis SBP: 150.3 ± 22.3 mmHg with low vs 166.6 ± 17.3 mmHg with standard dialysate sodium, *P* < .001; intradialytic SBP: 141.4 ± 18.0 mmHg with low vs 147.5 ± 13.6 mmHg with standard dialysate sodium, *P* = .034).

Similarly, SBP/DBP levels differed significantly between the two different dialysate sodium concentrations during the 44-h interdialytic interval, the first 24-h, first 20-h and second 24-h periods. Ambulatory SBP and DBP parameters during the 44-h interdialytic interval and the respective 24-h periods, with the low versus standard dialysate sodium concentration are also presented in Table 2. In addition, Supplementary data, Table S1 presents the differences between the two dialysate sodium interventions (low minus standard) for major BP indices for the overall period, the period before and the period after crossover.

Of importance, mean IDWG was significantly lower with low (1.4 ± 0.8 kg) than with standard dialysate sodium (1.8 ± 0.7 kg, *P* = 0.012). Pre-dialysis weight also showed a significant difference between low and standard dialysate sodium phase. Finally, the number of US-B lines pre-dialysis was also significantly lower with low [7 (4–14)] compared with standard dialysate sodium concentration [8 (6–13), *P* = .036].

### Ambulatory BP during day-time and night-time periods

Table 3 shows the BP levels during day-time and night-time periods of the different time intervals examined. As shown in the Table, ambulatory SBP levels during the whole 48-h, first 24-h and second 24-h day-time periods were significantly lower with low dialysate sodium. A similar pattern was evident for DBP, with significant differences observed during the various day-time periods with the low dialysate sodium concentration.

**Table 3:** Ambulatory BP levels during the various day-time and night-time periods of the 48-h interval with low vs standard dialysate sodium.

**Parameter**	**Low dialysate sodium**	**Standard dialysate sodium**	***P***
48-h interval			
Day-time SBP (mmHg)	138.1 ± 17.4	144.1 ± 14.8	**.002**
Day-time DBP (mmHg)	82.2 ± 14.2	85.0 ± 14.1	**.015**
Night-time SBP (mmHg)	135.8 ± 19.2	139.8 ± 15.4	.162
Night-time DBP (mmHg)	79.2 ± 13.7	81.4 ± 14.0	.122
First 24-h interval			
Day-time SBP (mmHg)	138.6 ± 18.5	145.4 ± 15.2	**.002**
Day-time DBP (mmHg)	83.0 ± 14.5	85.5 ± 14.6	**.022**
Night-time SBP (mmHg)	134.3 ± 21.4	139.6 ± 17.1	.082
Night-time DBP (mmHg)	78.2 ± 14.1	81.1 ± 14.8	**.032**
Second 24-h interval			
Day-time SBP (mmHg)	137.2 ± 16.7	142.6 ± 15.4	**.017**
Day-time DBP (mmHg)	81.0 ± 14.2	84.5 ± 14.0	**.008**
Night-time SBP (mmHg)	138.3 ± 18.3	140.2 ± 15.4	.564
Night-time DBP (mmHg)	81.1 ± 14.0	81.9 ± 14.2	.657

Data are presented as mean ± SD.

Night-time SBP during the 48-h, first 24-h and second 24-h periods were numerically, but not statistically, lower with low compared with standard dialysate sodium concentration. With regards to DBP, the lower BP levels observed during day-time periods with the low dialysate sodium were consistent across the various night-time periods, although only the difference during the first 24-h night-time was statistically significant.

No significant difference were observed in the dipping pattern (dippers, non-dippers and reverse dippers), during both the 1st 24-h (20.7%, 51.7% and 27.6% with low vs 10.3%, 72.4% and 17.2% with standard dialysate sodium, *P* = .714) and the 2nd 24-h period (6.9%, 41.4% and 51.7% with low vs 10.3%, 55.2% and 34.5% with standard dialysate sodium respectively, *P* = .167).

### Changes from baseline in peri- and intradialytic BP

**Figure 3:**
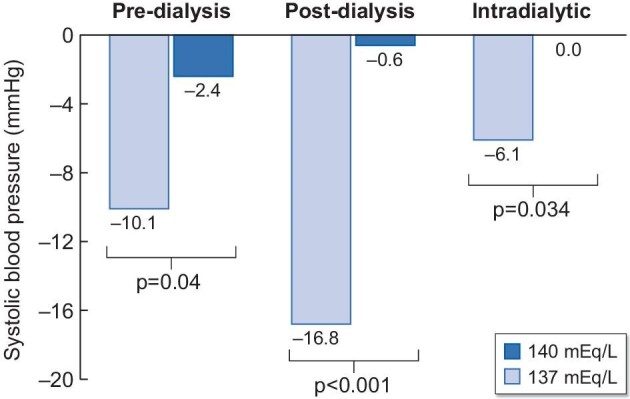
Comparison of changes from baseline in pre-dialysis, post-dialysis and intradialytic SBP with low versus standard dialysate sodium concentration.

The differences in the changes in pre-, post- and intradialytic BP from baseline between low and standard dialysate sodium concentrations are illustrated in Fig. 3. Mean change in intradialytic SBP/DBP was significantly greater with low dialysate sodium compared with standard dialysate sodium concentration (ΔSBP: –6.1 ± 12.8 mmHg with low vs 0.0 ± 14.1 mmHg with standard dialysate sodium, *P* = .034, ΔDBP: –2.5 ± 8.8 mmHg with low vs 0.7 ± 7.3 mmHg with standard dialysate sodium, *P* = .01). Reduction in post-dialysis SBP was significantly greater with low dialysate sodium (Δ post-dialysis SBP: –16.8 ± 26.4 mmHg with low vs –0.6 ± 17.0 mmHg with standard dialysate sodium, *P* < .001), while reduction in DBP was numerically but not statistically different between the two sodium concentrations. Of importance, a significant difference in the change in pre-dialysis SBP from baseline between the two study phases was noted (–10.1 ± 16.8 mmHg with low vs –2.4 ± 19.3 mmHg with standard dialysate sodium, *P* = .04).

### Associations of BP differences with baseline parameters of interest

**Figure 4:**
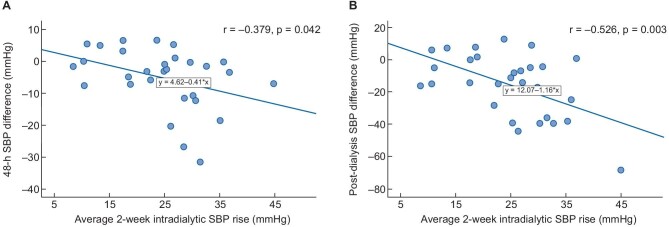
Correlations between (**A**) 48-h SBP difference and (**B**) post-dialysis SBP difference and average intradialytic SBP rise over the 2-week period before randomization.

As noted in Fig. 4, differences between interventions in 48-h SBP (r = –0.379, *P* = .042) and post-dialysis SBP (r = –0.526, *P* = .003) showed an inverse correlation with the rise in average intradialytic SBP over the 2-week period before randomization (Fig. 4). No correlations between differences in 48-h and post-dialysis SBP and baseline serum sodium (r = –0.044, *P* = .821; r = –0.040, *P* = .835), sodium gradient (r = –0.044, *P* = .821; r = –0.040, *P* = .835) and baseline total B-lines (r = –0.051, *P* = .792; r = –0.333, *P* = .078) were noted.

### Intradialytic symptoms and adverse events

One patient had one hypotensive episode (BP <90 mmHg) requiring intravenous fluid administration during the low dialysate sodium phase. No vascular-access thrombosis episode was evident during the study.

## DISCUSSION

This randomized crossover study examined the effect of low (137 mEq/L) versus standard (140 mEq/L) dialysate sodium concentration on ambulatory BP during a complete 48-h period in patients with IDH. Use of low dialysate sodium significantly reduced 48-h ambulatory SBP/DBP, with an observed mean difference of –5.3/–2.6 mmHg, compared with standard dialysate sodium. Low dialysate sodium also significantly reduced post-dialysis SBP/DBP (mean difference –16.2/–3.3 mmHg) and intradialytic SBP/DBP (mean difference –6.1/–3.1 mmHg). This pattern of lower BP with lower dialysate sodium was consistent across the different 24-h, day-time and night-time intervals examined, although the differences did not reach statistical significance for night-time. Pre-dialysis weight, IDWG and pre-dialysis B-lines assessed with lung US were also significantly lower with low compared with standard dialysate sodium.

The pathophysiology of IDH is multifactorial and not yet fully elucidated. Several mechanistic pathways are implicated in the pathogenesis of this phenomenon, including volume and sodium overload, overactivity of sympathetic nervous system and renin–angiotensin–aldosterone system, endothelial dysfunction,and increased arterial stiffness [4, 5]. Among them, volume overload is considered one of the most important. Earlier small studies showed that intensified ultrafiltration decreased BP and improved echocardiographic parameters in patients with IDH [26, 27]. In a cross-sectional study in 531 hemodialysis individuals, patients with increased post-dialysis BP had higher percentage of total and extracellular water, as assessed with bioimpedance spectroscopy compared with those with stable or decreased post-dialysis BP [28]. A case–control study including 18 patients with IDH and 18 controls demonstrated that IDH patients had higher ratio of extracellular water to body weight both before and after dialysis, as well as a higher ratio of extracellular water to total body water at the end of dialysis [12]. In a recent *post hoc* analysis of the Frequent Hemodialysis Network Daily Trial, bioimpedance estimated-shorter vector length (per 50 Ω/m) was associated with higher odds of IDH, confirming the above observations in a population performing frequent dialysis [29]. A positive sodium gradient during hemodialysis has been also suggested to play a key role in IDH. High concentration of dialysate sodium is associated with positive sodium balance and higher IDWG [30], while preliminary evidence suggested that high dialysate sodium may reduce endothelial NO release, leading to vasoconstriction and increased peripheral vascular resistance [31]. Finally, in a study including 206 hemodialysis patients, a linear positive association was identified between intradialytic SBP increase and dialysis sodium gradient [32].

Based on such observations, a few studies explored the effect of dialysate sodium concentration on BP levels in patients with IDH [33]. In a previous randomized crossover study in 16 patients with IDH, Inrig *et al*. showed that 1-week average intradialytic SBP was reduced by 9.9 mmHg with low (5 mEq/L lower than serum sodium) compared with high (5 mEq/L higher than serum sodium) dialysate sodium [13]. In a recent non-randomized interventional study, Nair *et al*. examined the effect of low (136 mEq/L) compared with standard (140 mEq/L) dialysate sodium over eight consecutive sessions in 50 patients with IDH [14]. Low dialysate sodium was associated with a significantly lower post-dialysis SBP/DBP. Another randomized crossover study recently evaluated the effect of individualized isonatremic (average of pre-dialysis serum sodium concentration) versus individualized hyponatremic (4 mEq/L below average of pre-dialysis serum sodium concentration) versus standard dialysate sodium of 140 mEq/L on peridialytic and 24-h BP in 11 patients with IDH [15]. Both isonatremic and hyponatremic dialysates were associated with lower pre- and post-dialysis SBP, but most importantly, hyponatremic dialysate also reduced mean 24-h SBP/DBP.

Our study confirms previous observations on significant reductions with low dialysate sodium of both post-dialysis and intradialytic SBP. These effects were recorded with a dialysate sodium level of 137 mEq/L (i.e. slightly higher than the average low dialysate levels of previous studies, ranging between dialysate sodium of 135 to 136.4) [13–15]. The intervention was well tolerated and safe, as indicated by the infrequent hypotensive episodes noted. SBP rise was not fully eliminated, but substantially attenuated with low dialysate sodium and a difference in mean intradialytic SBP of about 6 mmHg between the two interventions was observed; this effect was numerically lower than that in the study of Inrig *et al*. [13] probably due to the greater differences in dialysate sodium concentrations compared therein. Patients randomized to receive low-then-standard dialysate sodium (Group A) had greater BP drops with low sodium compared with patients who received standard-then-low dialysate sodium. This is consistent with the observations from Inrig *et al*. [13], suggesting the presence of carry-over effect in both studies. In our case, this could be, at least partially, attributed to the use of the standard dialysate sodium during the washout period, which led to longer exposure of all participants to this concentration (higher than the baseline serum sodium levels in most participants) during the study.

Since most of the aforementioned studies evaluated the effect of different dialysate sodium concentrations on regular BP readings acquired during the hemodialysis process and only one included 24-h ABPM, this study additionally expands the above observations by providing information for the complete 48-h intra- and interdialytic interval. Mean 48-h ambulatory BP was significantly lower with low compared with standard dialysate sodium and this finding was consistent during the 44-h, first 24-h, first 20-h and second 24-h periods. This is particularly important, since previous studies using 48-h ABPM suggest that patients with IDH have a profile of significantly elevated BP during both the intra- and interdialytic intervals [18, 34, 35], supporting the hypothesis that the intradialytic SBP rise may reflect a background hypertensive state rather than an isolated phenomenon observed only during the dialysis session. Recently, we also showed that the association between IDH and cardiovascular events is not independent of the interdialytic BP profile, suggesting that the increased risk for adverse outcomes could be, at least partially, explained by increased interdialytic BP levels [19]. As such, this finding of a BP lowering effect that may persist further from the intradialytic to the interdialytic BP suggests that lowering dialysate sodium may be a promising strategy to improve overall BP control and adverse outcome risk in these individuals. Use of 48-h ABPM enabled us to identify minor points, i.e. the fact that the nadir of BP values in patients dialyzing at the morning shift occurred at around 10 a.m. on the non-dialysis day, a fact that could be relevant to morning antihypertensive drug receipt in combination with absence of dialysis.

Other findings of our study include the significantly lower IDWG and pre-dialysis weight with low compared with standard dialysate sodium. As discussed above, patients with IDH commonly have volume overload, while a positive intradialytic sodium balance is associated with BP elevation during hemodialysis [30, 32]. Although some studies have suggested that lowering dialysate sodium concentrations may reduce BP levels independently of IDWG and dry weight [14, 36, 37], others have also observed a parallel effect on both BP and IDWG. In an earlier study, de Paula *et al*. observed a decrease in IDWG, interdialytic thirst score and episodes of IDH with individualized compared with standard dialysate sodium in 27 IDH patients [38]. Subsequent studies examining hemodynamic effects of individualized sodium supported this finding and in a recent study by Radhakrishnan *et al*. [39], apart from the observed reduction in the number of IDH episodes, a significant decrease in IDWG and thirst score was evident during the individualized dialysate sodium phase. Our results are consistent with these observations demonstrating not only a significant decrease in IDWG but also a reduction in the number of US-B lines with lower dialysate sodium, a finding that further enhances the hypothesis of a volume/sodium-related underlying hypertension mechanism in this population.

This study has strengths and limitations. To our knowledge, this is the first study to investigate the effect of low versus standard dialysate sodium concentration on ambulatory BP during a 48-h period, covering a complete intra- and interdialytic period, in patients with IDH. It followed a randomized cross-over design with a careful power estimation, and included a sufficient washout period of 2 weeks to avoid carryover effects. It also had a low drop-out rate, as only one patient had invalid ABPM and refused to continue, accounting for 3.3% of the study population. The duration of each intervention period may be considered by some relatively short (four hemodialysis sessions); however, longer intervention periods would require longer washout periods and may increase the chance of patient drop-outs. In addition, we followed a single-blinded study design where assessors were unblinded, and this could have introduced certain bias; however, our main outcome, 48-h SBP, is traditionally considered as objective. Another possible limitation is inclusion of patients based on clinical assessment of dry weight, rather than a more objective method, i.e. bioimpedance or lung US; this however was a conscious decision, as these non-clinical tools are not widely applied in every country, and we intended to perform a study with largely generalizable results. We did not use individualized dialysate sodium for each patient and did not measure serum sodium in each phase of the study to detect changes in pre-dialysis sodium; we opted for a rather “conservative” dialysate concentration of 137 mEq/L to avoid intradialytic complications. Moreover, the use of standard dialysate sodium concentration of 140 mEq/L during the washout period inevitably led to longer exposure to standard dialysate sodium, which may have contributed to the observed carry-over effect. Both of the above, however, do rather not change the essence of our findings, as either lower dialysate sodium in the intervention arm, or an intermediate dialysate sodium for the washout period would rather lead to higher between-group differences.

In conclusion, this is the first study examining the effect of low compared with standard dialysate sodium concentration on interdialytic BP during a complete 48-h period in patients with IDH. We demonstrated that low dialysate sodium can effectively reduce not only intradialytic but also 48-h ambulatory BP, potentially through a volume-dependent mechanism as suggested by the lower IDWG and pre-dialysis weight. These findings support modification of dialysate sodium as a major non-pharmacologic approach for BP management in patients with IDH. Future clinical trials should explore other interventions for ambulatory BP reduction in this high-risk population.

## Supplementary Material

gfae104_Supplemental_File

## Data Availability

Access to trial data can be made available to researchers upon reasonable request to the corresponding author, respecting local and national General Data Protection Regulation regarding personal data handling.
