# Supplementary material for: Low dialysate sodium and 48-h ambulatory blood pressure in patients with intradialytic hypertension: a randomized crossover study
Source: Nephrol Dial Transplant. 2024 May 6;39(11):1900–10. doi: 10.1093/ndt/gfae104 (PMC11522792; doi:10.1093/ndt/gfae104)
Supplement: gfae104_Supplemental_File [file gfae104_supplemental_file.docx]

**SUPPLEMENTAL MATERIAL**

**Title: Effect of low versus standard dialysate sodium on 48-h ambulatory blood pressure in patients with intradialytic hypertension: a randomized crossover study.**

**Running head:** Effect of low dialysate sodium on intradialytic hypertension.

**Authors:** Fotini Iatridi^1^, Konstantinos Malandris^2^, Robert Ekart^3^, Efstathios Xagas^4^, Antonios Karpetas^5^, Marieta P. Theodorakopoulou^1^, Artemios Karagiannidis^1^, Areti Georgiou^1^, Aikaterini Papagianni^1^, Pantelis Sarafidis^1^

**Affiliations:** 1) First Department of Nephrology, Hippokration Hospital, Aristotle University of Thessaloniki, Greece; 2) Clinical Research and Evidence-Based Medicine Unit, Aristotle University of Thessaloniki, Thessaloniki, Greece; 3) Department of Nephrology, University Clinical Centre Maribor, Maribor, Slovenia; 4) Frontis Dialysis Center, Athens, Greece; 5) Therapeutiki Hemodialysis Unit, Thessaloniki, Greece

**Correspondence:** Iatridi Fotini, MD, MSc, First Department of Nephrology, Hippokration Hospital, Aristotle University of Thessaloniki, Konstantinoupoleos 49, 54642, Thessaloniki, Greece. Tel: +30 6978995896. E-mail: fotini.iatridi@gmail.com

**Supplemental Table 1.** Differences between the two dialysate sodium concentrations (low minus standard) for major BP indices for the overall period, the period before crossover (1^st^ study period, when group A received low and group B received high dialysate sodium) and the period after crossover (2^nd^ study period when group A received high and group B received low dialysate sodium).

|  | **48-h**  **SBP Diff** | **48-h**  **DBP Diff** | **Pre-dialysis SBP Diff** | **Pre-dialysis DBP Diff** | **Post-dialysis SBP Diff** | **Post-dialysis DBP Diff** | **Intradialytic**  **SBP Diff** | **Intradialytic**  **DBP Diff** |
| --- | --- | --- | --- | --- | --- | --- | --- | --- |
| **Overall**  **(low minus standard)** | -5.3±9.5 (p=0.005) | -2.6±4.8  (p=0.07) | -7.8±19.3 (p=0.40) | -2.8±9.8  (p=0.135) | -16.2±9.5  (p<0.001) | -3.3±11.5  (p=0.134) | -6.1±14.7  (p=0.034) | -3.1±6.1  (p=0.013) |
| **Before crossover**  **(low minus standard)** | -17.8±5.8 (p=0.005) | -4.1±5.6  (p=0.469) | -10.8±6.7 (p=0.117) | -0.8±4.4  (p=0.862) | -26.2±7.3  (p=0.001) | -6.6±5.9  (p=0.271) | -20.1±5.5  (p=0.001) | -6.2±5.2  (p=0.242) |
| **After crossover (low minus standard)** | 6.6±5.1 (p=0.208) | -1.4±4.9  (p=0.784) | -5.0±5.5 (p=0.375) | -4.9±4.1  (p=0.242) | -6.3±7.3  (p=0.394) | -0.6.±5.2  (p=0.913) | 6.7±5.3  (p=0.215) | -0.6±4.5  (p=0.892) |
| **Randomization sequence**  **(A vs B)⃰** | -6.0±3.6  (p=0.120) | -3.0±4.3  (p=0.095) | -2.7±7.3  (p=0.719) | -1.2±3.7 (p=0.746) | -5.2±7.4 (p=0.485) | -5.6±4.3  (p=0.202) | -12.1±5.1  (p=0.025) | -5.8±2.0  (p=0.008) |

⃰differences between the two study groups of randomization sequence (A vs B) in the differences of various BP indexes between the two interventions (low minus standard)

**Supplemental Figure 1.** Time periods of the 48-h recording for the different outcomes studied (data displayed for patients in the morning shifts).


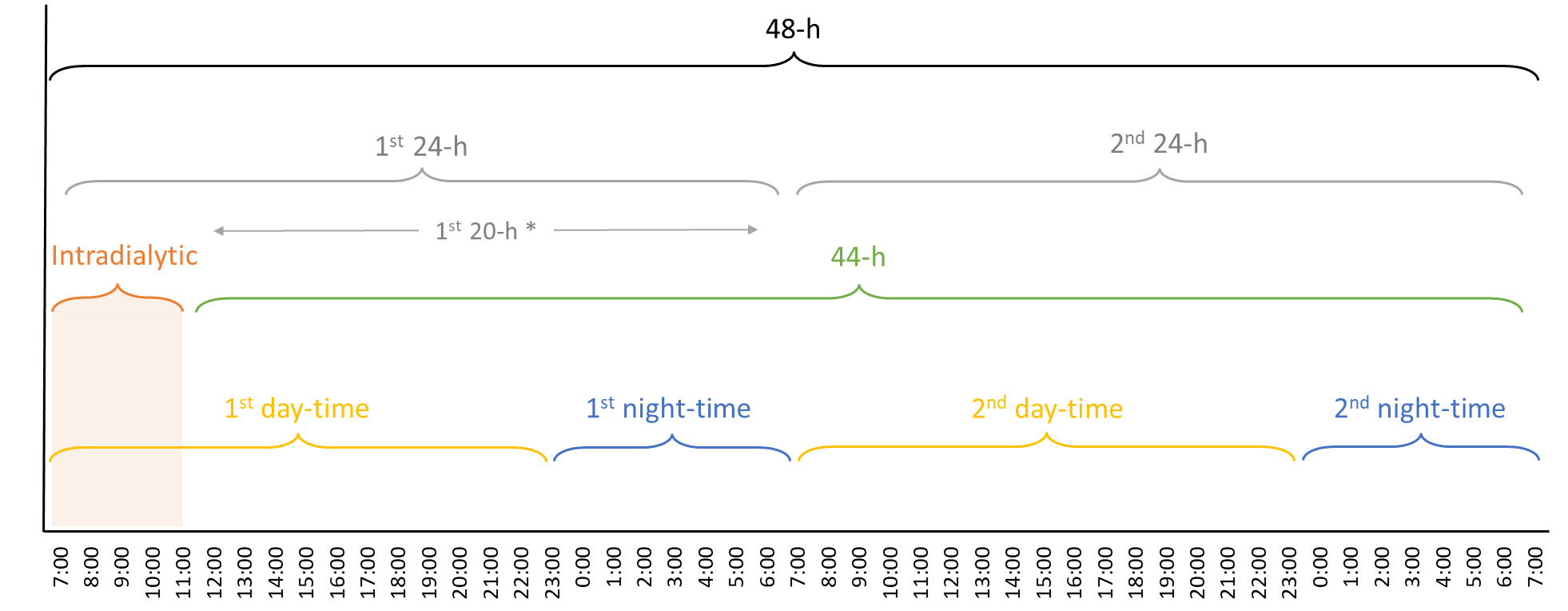


⃰ 1^st^ 20-h: 1^st^ 24-h without the 4-h dialysis session (1^st^ 24-h minus intradialytic)

48-h day- and night-time periods are defined as the average of the 1^st^ and 2^nd^ day- and night-time periods, respectively
